# Demographic profile of patients and risk factors associated with suicidal behaviour in a South African district hospital

**DOI:** 10.4102/safp.v63i1.5330

**Published:** 2021-10-28

**Authors:** Aneeth Sadanand, Selvandran Rangiah, Rolan Chetty

**Affiliations:** 1 Department of Family Medicine, College of Health Science, University of KwaZulu-Natal, Durban, South Africa; 2 Department of Family Medicine, Northdale Hospital, Pietermaritzburg, South Africa

**Keywords:** suicidal behaviour, demographical profile, risk factors, district hospital, urban

## Abstract

**Background:**

Suicidal behaviour comprises self-destructive thoughts coupled with attempts at suicide, which negatively impacts the patient, family, friends, and their community. There is a paucity of data on factors influencing suicidal thoughts and behaviour in South Africa. The aim of this study was to evaluate demographic profile and risk factors associated with suicidal behaviour.

**Methods:**

In this retrospective descriptive and observational study, 282 medical records of patients with suicidal behaviour were studied. The risk factors and age at occurrence were tabulated. Descriptive analyses were undertaken to understand how they were distributed across key socio-demographic groups.

**Results:**

Suicidal behaviour was particularly prominent amongst the female population. The suicidal ideation, plan and non-fatal suicide were reported by 48.6%, 29.1% and 36.5%, of patients respectively. The prevalence for suicidal ideation was significantly higher in females (54.5% vs. 31.5%; *p* < 0.0007) but not for suicidal plan (28.7% vs. 30.1%; *p* < 0.81) and suicidal attempt (37.3% vs. 34.2%; *p* = 0.63) as compared with males. Suicidal behaviour was positively associated with depression (*r* = 0.56, *p* < 0.001) and negatively associated with age (r = −0.16, *p* = 0.01). Multivariate logistic regression analysis revealed that suicidal behaviour was influenced by female gender, poor social support, depression and a family history of non-fatal suicide.

**Conclusion:**

This research has confirmed an association between female sex and factors associated with a higher risk of suicidal behaviour.

## Introduction

Suicidal behaviour describes a spectrum of negative thoughts and includes non-fatal suicides with varying degrees of intent and lethality, which may lead to (fatal) suicide.^1^ Suicidal behaviour is caused by an imbalance between factors that increase distress and decrease restraint.^1^ It is estimated that more than 800 000 suicides occur every year, with 79% occurring in low- and middle-income countries.^2^ Furthermore, it is estimated that there is a 25:1 ratio between non-fatal suicides and fatal suicides.^3^ A past non-fatal suicide attempt is perhaps the best indicator that a patient is at increased risk of suicide.^4^ Risk factors for suicide may be both individual and familial.^5^ Familial risk factors may be caused by family history of suicidal behaviours^6^ and familial or genetic associations with suicidality.^7^ Individual risk factors for suicide include high levels of hopelessness, poor problem-solving skills and a history of aggressive behaviour.^5^ In addition, exposure to suicidal behaviour and thoughts may be associated with increased risk of suicide, non-fatal suicide and suicidal ideation.^8,9^

In order to obtain accurate data of suicidal behaviour amongst the population of South Africa, the use of a systematic data collection model from the various provinces may be beneficial towards gathering information and occurrences of patients presenting with suicidal behaviour.

In the South African context, previous studies on suicidal behaviour are scarce probably because it is assumed that suicidal behaviour occurs less frequently in the local communities.^10^ More recent studies have shown that suicidal behaviour amongst the youth has increased significantly in parts of Africa including South Africa.

Du Toit et al^11^ found that of the 258 patients who presented with suicidal behaviour in Bloemfontein, majority were female (68.9%). Possible risk factors contributing to suicidal behaviour are financial difficulties, interpersonal relationship problems and mental health illnesses.^11^ Mental health issues, past history of deliberate self-harm, poor self-esteem and feeling of hopelessness are seen amongst patients presenting with non-fatal suicide.^12^ Patients likely to cause deliberate self-harm have reported financial stress, interpersonal relationship problems, lack of tertiary education and past psychiatric illness as the main reasons contributing to suicidal behaviour.^13^ The current increasing trend of patients presenting with non-fatal suicide in South Africa will further impact the nation’s healthcare services and place added strain on the resources of South Africa.^12^

Non-fatal suicide patients who present to a healthcare establishment frequently require medication, counselling, a social worker, psychology and psychiatric evaluation and therapy.^14^ Social workers provide an important role towards determining and addressing risk factors for suicidal behaviour. Social workers assist in patient and family counselling and aid patients to fulfil their basic needs including housing, access to food, social services and healthcare. Kinyanda et al (2009)^15^ reported suicide rates of 15–20 per 100 000 from 2005 to 2007 in northern Uganda. Studies in sub-Saharan Africa have methodological difficulties such as data compilation, poor research designs, assessment instruments together with poor research infrastructure and collaboration not being practised. In South Africa, divergent cultural and religious perceptions of suicidal behaviour have also influenced the assumption that suicidal behaviour is not a significant problem in parts of Africa.^16^ Despite an increase in research investigating suicidal behaviour in Africa, the rates are still likely to be under-reported and a good understanding of the full burden of suicidal behaviour is limited. This is largely due to a lack of research infrastructure and funds, a lack of expertise in suicide research and inadequate inter-African research collaborations. Furthermore, limited and outdated studies as well as a lack of standardised research designs and assessment instruments also contribute to the under reporting of data.^17^ Suicidal behaviour in most parts of Africa still carries negative cultural sanctions, therefore, perpetuating non-reporting.^18,19^

Suicidal behaviour and its consequences are a global threat with an estimated 60% increase over a couple of decades.^20^ As a major health problem in South Africa, it is estimated that about 6500 suicides and 130 000 non-fatal suicides occur annually within the country, with at least one suicide taking place every 40 s, compared with one non-fatal suicide every 3 s.^20^ The suicide rates in South Africa range from 11.5 to 25.0 per 100 000 of the population.^21^ Approximately 11.0% of all non-natural deaths in South Africa are as a result of suicide.^21^ Furthermore, limited and outdated studies and a lack of standardised research designs and assessment instruments also contribute to the under-reporting of data.^17^

Suicidal behaviour in most parts of Africa also still carries negative cultural sanctions therefore perpetuating non-reporting.^17^

Research on the profile of suicidal behaviour amongst patients in South Africa will aid in understanding the magnitude of the problem, the specific risk factors in the population and how management strategies may be improved. The aim of this study was to evaluate the demographic profile and risk factors associated with suicidal behaviour.

## Material and methods

### Study design

This study was a retrospective descriptive and observational record review study of the demographic, socioeconomic data of patients who presented to the Department of Family Medicine at a district hospital for management of suicidal behaviour between the study period of 01 July 2018 and 31 December 2018.

### Study population and sample size

This was a descriptive study and therefore a sample size calculation was not necessary. The study population included medical records of 282 patients with a diagnosis of suicidal behaviour who presented to the emergency department and were admitted to the Department of Family Medicine at Northdale district hospital for management over a study period of six months. The study population eligible for this study was inpatients with suicidal behaviour; patients with fatal suicide were excluded.

### Data collection and data collection tool

A preformed data abstraction tool was used to capture required data from clinical medical records of the patients diagnosed with suicidal behaviour over a study period of six months. The chart reviews were performed by the principal author. A predesigned data tool was used to obtain detailed information on patients’ ages. The abstraction tool included baseline demographic data (age, sex, marital status) and clinical characteristics (risk factors for suicidal behaviour). The names and hospital number of the participants who were admitted for suicidal behaviour were obtained from the medical registry. The data sheet was checked for completeness and consistency by the principal investigator and the supervisor. The data sheet was pretested on 10 healthcare workers working in allied health facilities. Findings from the pre-test were used to modify the data sheet to clarify the wordings. Minimal changes were required to the data sheet, re-piloting was not necessary. All suicidal behaviour patients who were 15 years old or above were included and those under the age of 15 and those patients admitted for other medical reasons were excluded. Risk factors were documented in a structured format. Data from the collection sheets were entered into an excel spreadsheet.

### Variables of the study

**The dependent variable of the study was suicidal behaviour. The independent variable comprised:** socio-demographic variables: sex (male female), social support, depression, family history of suicidal attempt.

### Statistical analysis

After the data had been checked for completeness and accuracy, it was coded manually and then entered into excel spreadsheet and exported to Statistical Package of Social Science (SPSS) version 27 for analysis. Descriptive statistics was performed on numerical value, mean, standard deviation, frequencies, proportion to describe study population in relation to dependent and independent variables. A Pearson’s correlation was performed between suicidal ideation with depression and age. A multivariable logistic regression was performed and a *p* < 0.05 with 95% confidence interval (CI) for odds ratio (OR) was used to determine significance.

## Results

A total of 282 medical records of patients who presented to Northdale Hospital with suicidal behaviour from the 01 July 2018 to 31 December 2018 were reviewed. Of these total 282 patients, 217 (77%) patients had presented for the first time whilst 65 (23%) presented with a previous history of suicidal behaviour. In all 209 (74.1%) of the study population were females. Suicidal behaviour was observed amongst the study population across all age groups and the effect was particularly prominent amongst women aged 15–25 and males aged 15–35 years. Most of the study population were single (48.2%), more than 40% were in a stable relationship and 10.6% were married (Table 1).

**TABLE 1:** Age groups of the study population based on gender.

Variable	Men (*n* = 73)		Women (*n* = 209)		Total (*N* = 282)	
	*n*	%	*n*	%	*n*	%
**Age groups**						
15–25	24	32.9	116	55.5	140	49.65
26–35	29	39.7	45	21.5	74	26.2
36–45	13	17.8	20	9.6	33	11.7
46–55	4	5.5	21	10.0	25	8.9
56–65	2	2.7	6	2.95	8	2.8
> 65	1	1.4	1	0.5	2	0.7
**Marital status**						
Single	22†	16.9	108†	83.1	130‡	46.1
Cohabiting	36§	31.0	80§	69	116‡	41.1
Married	9¶	30.0	21¶	70	30‡	10.6

† , *N* = 130;

‡ , *N* = 282;

§ , *N* = 116;

¶ , *N* = 30.

### Prevalence of suicidal behaviour

Sex (male or female) based differences with respect to various predisposing variables to suicidal behaviour have been listed in Table 2.

**TABLE 2:** Gender differences in the development of suicidal behaviour.

Warning signs for suicidal behaviour	Total	Female		Male		*p*
		*n* †	%	*n* ‡	%	
Family history of suicide	19	14	6.7	5	6.8	0.9700
Chronic illness	47	33	15.8	14	19.2	0.5000
Psychiatric illness	42	32	15.3	10	13.7	0.7400
Poor social support	139	103	49.3	36	49.3	1.0000
Substance abuse	93	54	25.8	39	53.4	0.0001*
Sexual and/or physical abuse	43	31	14.8	12	16.4	0.7400
Depressed	142	116	55.5	26	35.6	0.0020*
Positive psychotic symptoms	28	20	9.6	8	10.9	0.7400
Hopelessness	171	131	62.7	40	54.8	0.2300
Worthlessness	196	150	71.8	46	63.0	0.1500
Anhedonia	130	103	49.3	27	36.9	0.0600
Anxiety	56	28	13.4	28	38.4	0.0001*
Panic attacks	29	25	11.9	4	5.5	0.1500
Impulsivity	120	90	43.1	30	41.1	0.7600
Anger	186	138	66.0	48	65.8	0.9000
Recent substance use	143	101	48.3	42	57.5	0.1700
Suicidal ideation	137	114	54.5	23	31.5	0.0007*
Suicidal intent	103	78	37.3	25	34.2	0.6300
Suicidal plan	82	60	28.7	22	30.1	0.8100
Access to lethal means	109	73	34.9	36	49.3	0.0200*
Past suicidal behaviour	65	54	25.8	11	15.1	0.0600
Current problems seem unsolvable	171	134	64.1	37	50.7	0.0400*
Suicide violent command hallucinations	19	9	4.3	10	13.7	0.0050*

* , Significant values at *p* < 0.05.

† , *n* = 209;

‡ , *n* = 73.

### Correlation studies

Pearson’s correlation studies showed that suicidal behaviour was positively associated with depression (*r* = 0.56, *p* < 0.001) and negatively associated with age (*r* = −0.16; *p* = 0.01).

As shown in Table 3, in multivariate logistic regression analysis revealed that the odds of suicidal behaviour were higher amongst the female gender (adjusted odds ratio [AOR]: 1.54; 95% CI: 1.01–2.36). As shown in Table 3 the crude odds ratio (COR) for poor social support was (COR: 3.02; 95% CI: 2.46–4.69), depression (COR: 2.46; 95% CI: 1.95–3.58) and for family history of non-fatal suicide (COR: 1.78; 95% CI: 1.02–2.58).

**TABLE 3:** Odds ratio of suicidal behaviour.

Study variable	Suicidal behaviour (*N* = 282)				COR	AOR	95% CI	*p*
	Yes		No					
	*n*	%	*n*	%				
**Gender**							2.14–5.23	0.0001
Male	73	25.9	209	74.1	Ref	-		
Female	209	74.1	73	25.9	3.32	1.54		
**Social support**							2.46–4.69	0.0004
Good	139	49.3	36	12.8	Ref	-		
Poor	70	24.8	37	13.1	3.02	-		
**Depression**							1.95–3.58	0.0001
Yes	116	41.1	26	2.8	2.46	-		
No	93	32.9	47	16.7	Ref	-		
**Family history of suicidal attempt**							1.02–2.58	0.0400
Yes	14	4.9	5	1.8	1.78	-		
No	195	69.1	68	24.1	Ref	-		

COR, crude odds ratio; AOR, adjusted odds ratio; CI, confidence interval.

## Discussion

The factors associated with suicidal behaviour in this study were female sex, poor social support, anxiety, substance abuse and a history of depression. Suicidal behaviour was observed amongst the study population across all age groups and the effect was particularly prominent amongst women aged 15–25 years and men aged 15–35 years.

Gender-specific differences in suicidal behaviour have been analysed in a number of studies with conflicting outcomes.^21,22,23,24,25^ A number of studies addressing sex (male or female) differences in suicidal behaviour have reported no significant differences between males and females,^26^ whilst others have confirmed significant relationships between suicidal behaviour and sex (male or female).^27,28,29^

In a more recent study, no significant sex-based difference was evident.^30^ In this study however, sex (male or female) affected the prevalence of suicidal behaviour. In South Africa a recent systematic multicentre European study^32,33^ on parasuicide showed that young adults in the age group 16–25 years of age were more vulnerable^31^ compared with the age group of 15–34 years. Similar studies elsewhere have reported young people as target group with respect to parasuicide.^34,35^

The OR of a female displaying suicidal behaviour was three times higher compared with a male in this study, which is in accordance with the results of other published studies in different settings.^36,37^ The discrepancy might be explained on the basis of the gender role socialisation theory, which states that females are expected to be dependent and indecisive, and express their stress via rumination leading to a higher rate of non-fatal suicides.^38^ In a study in South Africa, a risk for suicidal behaviour was observed amongst the age group of 18–34 years, females and less educated.^39^ Zitzow and Desjarlait in Zambia found amongst adolescents that the sex of the study population, feelings of sadness or hopelessness the use of alcohol and marijuana were important variables in suicidal behaviour.^40^

Our results did support our hypothesis that females would demonstrate a higher frequency of suicidal behaviour compared with males. In line with our other hypotheses, our results showed a significant sex difference between age groups for suicide behaviour, where in most of the age groups female suicidal behaviour was more frequent compared with males.

Consistent with previous studies,^9,41^ this study has found that female sex and, in unadjusted analysis, those unemployed within the ages of 15–65 year were associated with suicidal ideation, plans or attempts. In an earlier study conducted locally the prevalence of suicidal ideation was 9.1%, plans were 3.8% and attempts were 2.9%. In contrast, the suicidal ideation, plan and attempt reported in our study were at 48.6%, 29.1% and 36.5%, respectively. The prevalence of suicidal ideation was significantly higher amongst female than male study population (54.5% vs. 31.5%; *p* < 0.0007), whilst the prevalence of suicidal plan (28.7% vs. 30.1%; *p* < 0.8100) presented the same amongst both. However, suicidal attempts were higher amongst the male than the female study population (37.3% vs. 34.2%; *p* = 0.61). In a questionnaire-based study involving 874 (females [63.1%] and males [36.9%]) medical students aged < 25 years (94.1%), a high prevalence of suicidal ideation (32.3%) and suicidal attempt (6.9%) was found, which is three times higher than the general age-appropriate South African population.^42^ Consistent with previous studies,^11,43^ this study has found that female sex and, in unadjusted analysis, those unemployed within the ages of 15–65-years were associated with suicidal ideation, plans or attempts.

It has been reported that females were more likely to have suicidal ideation and attempted suicide than male.^9^ In Bhutan, around 3% of the adults aged 18–69 years reported having suicidal ideation and 0.7% ever attempted suicide. The prevalence is slightly higher than that of the 12-month global estimate of 2.1% for suicidal ideation and 0.4% for non-fatal suicides in developing countries.^44^ In Ethiopia, the suicidal ideation was reported as 7.0%, plan 4.6% and suicide attempt 3.7%.^44^ In South Africa, the prevalence of lifetime non-fatal suicide was 2.9%.^45^ The high rate of suicidal behaviour amongst adolescents in Zambia is also confirmed in a previous study , with a prevalence of past 12-month suicidal ideation of 31.1%.^46^ The suicide rate in Zambia (11.3 per 100 000) was similar to the rate in lower middle-income countries (11.4 per 100 000).^47^ In this study, we found female gender, being single, being unemployed and having a family history of suicide were independently associated with having suicidal ideation.

A significant sex difference was found only for suicidal ideation indicating a higher rate amongst female study population (*n* = 114; 54.5%) as opposed to the male study population (*n* = 23; 31.5%; *p* = 0.0007). Voss et al reported that a significant sex difference was found only for suicidal plan but not for suicidal ideation and suicidal attempt, indicating a higher rate amongst female participants compared with male participants.^48^ In contrast, this study found a significant sex difference only for suicidal ideation and not for suicidal plan and attempt.

The results for suicidal ideation were mixed. The increasing trend in suicidal ideation does not by itself reflect an increasing risk of suicide compared with the trends found for intentional self-harm.^48^ However, this research also suggested that the risk of suicide following suicidal ideation may be mediated by other factors, such as psychiatric comorbidity. In a more recent study from Eswatini it was found that female sex, childhood sexual abuse, adult sexual abuse, threats, death of a family member from suicide and family alcohol problems increased the odds and prevalence in the past 12 months for suicidal ideation, suicidal plans or even non- fatal suicide.^49^ It is essential to note that risk factors for suicidal ideation, plans and/or attempts may include people with chronic illness, such as epilepsy and cardiovascular disease, personality factors, unemployment and lower socioeconomic status, childhood adversity, adverse life events, female sex, younger age and family history of suicide.^9,50,51^

One of the predictors of suicidal thoughts in this study was poor social support. The study population who had poor social support were 3.02 times more likely to have suicidal thoughts compared with study population who had good social support. A recent study from Ethiopia reported that participants with poor social support were 1.66 times more likely to have suicidal thoughts compared with participants who had strong social support.^52^ A substantial number of this study population reported previous history of suicidal thoughts. In this regard, the female study population outnumbered male study population. Similar findings were reported in studies carried out locally.^8,20,53^

Globally, suicide and human immunodeficiency virus/acquired immunodeficiency syndrome (HIV/AIDS) remain two of the greatest healthcare issues, particularly in low- and middle-income countries where approximately 85% of suicides occur.^54,55^ South Africa has the highest rates of HIV in the world. The strong connection between HIV/AIDS and suicide is a major concern in South Africa, due to the soaring HIV rate in the country; and particularly among younger adults.^56^ Unfortunately, the data on the HIV status of our participants were not captured. The high correlation between physical and mental illness and suicidal behaviour highlights the importance of early screening, detection, treatment and management of these illnesses. While several studies have investigated the risk factors for suicidal behaviour, very little emphasis has been placed on identifying specific, contextual individual, family and community factors which may protect against the development of suicidal behaviour, especially in relation to young adults. A recent study conducted during the COVID-19 pandemic have revealed risk factors for suicidal behaviour which included high levels of stress, anxiety, and depression in the community.^57,58^

Factors positively associated with suicidal thoughts in our study were female gender, poor social support, anxiety, substance abuse and depression. Family history of suicide did not influence suicidal behaviour amongst our study population. In another study, the factors positively associated with suicidal thoughts included female gender, poor social support, not frequently engaging in religious practice, family history of non-fatal suicide, rural residence and lifetime alcohol use.^52^ Suicidal behaviour has been shown to be associated with a number of risky behaviours that in turn may also have immediate and long-term adverse health consequences.

It may be assumed that suicidal behaviour is even more prevalent in the general population than what the results of this study suggest. The presence of a mental disorder (e.g. depression or substance use disorder) during an interview about suicidal behaviour could have had an effect on the recall of suicidal episodes, although so far little is known about the direction of this effect across different psychopathologic disorders. The information assessed in this study was based on retrospective reports, thus it may be affected by recall bias.

It is important to note that this study did not measure suicide rates but only the rate of reports of suicidal behaviour. This, in itself, is an interesting topic for further investigation but was not the subject of our study. In addition, this study because of the non-availability of relevant information did not examine some important suicidal factors, such as body weight, physical activity, gender-based violence, domestic violence and marital problems, and smoking. Future studies may benefit by doing a prospective study, ensuring detailed examination on these remaining suicidal factors and obtaining expertise, if necessary.

### Limitation of study

The retrospective nature of this study was its main limitation, together with the drawbacks of poor documentation and missing information. The reporting of suicide information can be very sensitive because of the associated social stigma that could have led to under-reporting of suicide information. Thus, the prevalence of suicidal ideation, suicide plan and non-fatal suicide may be underestimated.

## Conclusion

In conclusion, this research has confirmed an association between female gender and factors associated with a higher risk of suicidal behaviour following hospitalisation.

### Recommendations

The findings of this study can be useful for policymakers in designing appropriate strategies for the prevention of suicidal behaviours. Non-fatal suicides are common and constitute a serious problem for public health, thus it is very important to evaluate risk factors for suicidal behaviour to plan strategies for its monitoring and prevention, as well as intervention.

## References

[1] Leadholm AK, Rothschild AJ, Nielsen J, et al. Risk factors for suicide among 34,671 patients with psychotic and non-psychotic severe depression. J Affect Disord. 2014;156:119–125. 10.1016/j.jad.2013.12.003 24388683

[2] World Health Organization (WHO). Suicide [homepage on the Internet]. 2019 [cited 2020 Sep 5]. Available from: https://www.who.int/news-room/fact-sheets/detail/suicide

[3] Nock MK, Hwang I, Sampson N, et al. Cross-national analysis of the associations among mental disorders and suicidal behavior: Findings from the WHO World Mental Health Surveys. PLoS Med. 2009;6(8):e1000123. 10.1371/journal.pmed.1000123 19668361 PMC2717212

[4] Olfson M, Shafef D, Marcus SC, et al. Relationship between antidepressant medication treatment and suicide in adolescents. Arch Gen Psychiatry. 2003;60(10):978–982. 10.1001/archpsyc.60.9.978 14557142

[5] Gould MS, Greenberg T, Velting DM, et al. Youth suicide risk and preventive interventions: A review of the past ten years. J Am Acad Child Adolesc Psychiatry. 2003;42(4):386–405. 10.1097/01.CHI.0000046821.95464.CF 12649626

[6] Apter A, Gvion Y. Adolescent suicide and attempted suicide. In: Wasserman D, editor. Suicide: An unnecessary death. 2nd ed. London: Oxford University Press, 2016; p. 5–12.

[7] Sokolowski M, Wasserman J, Wasserman D. An overview of the neurobiology of suicidal behaviors as one meta–system. Mol Psychiatry. 2015;20(1):56–71. 10.1038/mp.2014.101 25178164

[8] Hill NTM, Robinson J, Pirkis J, et al. Association of suicidal behavior with exposure to suicide and non-fatal suicide: A systematic review and multilevel meta-analysis. PLoS Med. 2020:17(3):e1003074. 10.1371/journal.pmed.1003074 32231381 PMC7108695

[9] Dendup T, Zhao Y, Dorji T, Phuntsho S. Risk factors associated with suicidal ideation and non fatal suicides in Bhutan: An analysis of the 2014 Bhutan STEPS Survey data. PLoS One. 2020;15(1):e0225888. 10.1371/journal.pone.0225888 31999708 PMC6991943

[10] Schlebusch L. Depression and suicidal behaviour. S Afr Fam Pract. 2005;47(5):61–63. 10.1080/20786204.2005.10873234

[11] Du Toit EH, Kruger JM, Swiegers SM, et al. The profile analysis of attempted-suicide patients referred to Pelonomi Hospital for psychological evaluation and treatment from 1 May 2005 to 30 April 2006. S Afr J Psychiatr. 2008;14(1):20–26. 10.4102/sajpsychiatry.v14i1.40

[12] Moosa Y, Jeenah Y, Pillay A, Vorster M, Liebenberg R. Non-fatal suicidal behaviour at the Johannesburg General Hospital. Afr J Psychiatr. 2005;8(3):104–107. 10.4314/ajpsy.v8i3.30192

[13] Van Zyl PJ, Bantjes J, Breet E, Lewis I. Motives for deliberate self-harm in a South African tertiary hospital. S Afr J Psychiatr. 2021;27:1524. 10.4102/sajpsychiatry.v27i0.1524 33604071 PMC7876963

[14] Nakin D, Joubert G, Pretorius PJ, Van Vuuren MJV. Evaluation of attempted-suicide management in a rural district of KwaZulu-Natal. S Afr J Psychiatry. 2007;13(2):52–55. 10.4102/sajpsychiatry.v13i2.28

[15] Kinyanda E, Nakku J, Oboke H, Oyok T, Ndyanabangi S, Olushayo O. Suicide in rural war affected Northern Uganda: A study from 4 sub-counties. Paper presented at the XXV World Congress on suicide prevention of the International association for suicide prevention 2009 October 27–31 at Montevideo, Uruguay.

[16] Schlebusch L. Suicide prevention: A proposed national strategy for South Africa. Afr J Psychiatry. 2012;15(6):436–440.https://doi.org/10.4314/ajpsy.v15i6.56 10.4314/ajpsy.v15i6.56 23160620

[17] Palmier JB. Prevalence and correlates of suicidal ideation among students in sub-Saharan Africa [thesis]. Atlanta: Faculty of Georgia State University; 2011.

[18] World Health Organization. Preventing suicide: A global imperative [homepage on the Internet]. 2014 [cited 2020 Sep 5]. Available from: https://www.who.int/mental_health/suicide-prevention/world_report_2014/en/

[19] Hawton K, Saunders KEA, O’Connor RC. Self-harm and suicide in adolescents. Lancet. 2012;379(9834):2373–2382. 10.1016/S0140-6736(12)60322-5 22726518

[20] Naidoo SS, Schlebusch L. Socio-demographic and clinical profiles of suicidal patients requiring admission to hospitals south of Durban. S Afr Fam Pract. 2013;55(4):373–379. 10.1080/20786204.2013.10874379

[21] Masango SM, Rataemane ST, Motojesi AA. Suicide and suicide risk factors: A literature review. S Afr Fam Pract. 2008;50(6):25–28. 10.1080/20786204.2008.10873774

[22] Denning D, King D, Cox C. Method choice, intent, and gender in completed suicide. Suicide Life Threat Behav 2000;30(3):282–288. 11079640

[23] Moscicki EK. Gender differences in completed and attempted suicides. Ann Epidemiol. 1994;4(2):152–158. 10.1016/1047-2797(94)90062-0 8205283

[24] Aghanwa H. The determinants of attempted suicide in a general hospital setting in Fiji Islands: A gender-specific study. Gen Hosp Psychiat. 2004;26(1):63–69. 10.1016/j.genhosppsych.2003.07.003 14757305

[25] Kumar CTS, Mohan R, Ranjith G, Chandrasekaran R. Gender differences in medically serious non-fatal suicides: A study from South India. Psychiatry Res. 2006;144(1):79–86. 10.1016/j.psychres.2005.11.012 16919336

[26] Bongongo T, Nkuinika K, Nzaumvila DK, et al. Profile of parasuicide cases attending Brits Hospital, North West province, South Africa: A 5- year chart review. J Hum Ecol. 2020;72(1–3):276–282. 10.21203/rs.3.rs-56437/v1

[27] Platt S, Bille-Brahe U, Kerkhof A, et al. Parasuicide in Europe: The WHO/EURO multicentre study on parasuicide. Part I: Introduction and preliminary analysis for 1989. Acta Psychiatr Scand. 1992;85(2):97–104. 10.1111/j.1600-0447.1992.tb01451.x 1543046

[28] Pfaff JJ, Acres J, Wilson M. The role of general practitioners in parasuicide: A Western Australia perspective. Arch Suicide Res. 1999;5(3):207–214. 10.1080/13811119908258330

[29] Shreeda S, Tawka N, Mohammeda N, Elmahdib M. Toxic agents used for parasuicide in Damietta Governorate, Egypt. Middle East Curr Psychiatry. 2011;18(1):11–17. 10.1097/01.XME.0000392843.71735.9d

[30] Berg HVD, Tancread H, Low D. Adolescents’ perceptions of stressors contributing to suicidal behaviour. S Afr J Soc Work Soc Develop [serial online]. 2014 [cited 2021 Jun 4];6(2). Available from: https://upjournals.co.za/index.php/SWPR/article/view/2243

[31] Miranda-Mendizabal A, Castellvi P, Pares-Badell O, et al. Gender differences in suicidal behaviour in adolescents and young adults: Systemic review and meta-analysis of longitudinal studies. Int J Public Health. 2019;64:265–283. 10.1007/s00038-018-1196-1 30635683 PMC6439147

[32] Freeman A, Mergl R, Kohls E, et al. A cross national study on gender differences in suicide intent. BMC Psychiatry. 2017;17:234. 10.1186/s12888-017-1398-8 28662694 PMC5492308

[33] Bagley CA, Shahnaz A, Simkhada P. High rates of suicide and violence in the lives of girls and young women in Bangladesh: Issues for feminist intervention. Soc Sci. 2017;6(4):140. 10.3390/socsci6040140

[34] King TL, Shields M, Sojo V, et al. Expressions of masculinity and associations with suicidal ideation among young males. BMC Psychiatry. 2020;20:228. 10.1186/s12888-020-2475-y 32398056 PMC7218581

[35] Shafti SS. Suicide and non-fatal suicides among psychiatric patients: A contrast between adults and adolescents. MOJ Clin Med Case Rep. 2020;10(1):25–29. 10.15406/mojcr.2020.10.00336

[36] Li ZZ, Li YM, Lei XY, et al. Prevalence of suicidal ideation in Chinese college students: A meta-analysis. PLoS One. 2014;9(10):e104368. 10.1371/journal.pone.0104368 25285890 PMC4186746

[37] Van Niekerk L, Scribante L, Raubenheimer PJ. Suicidal ideation and attempt among South African medical students. S Afr Med J. 2012;102(6):372–373. 10.7196/SAMJ.5503 22668910

[38] Borges G, Nock MK, Haro Abad JM, et al. Twelve-month prevalence of and risk factors for non-fatal suicides in the World Health Organization World Mental Health Surveys. J Clin Psychiatry. 2010;71(12):1617–1628. 10.4088/JCP.08m04967blu 20816034 PMC3000886

[39] Joe S, Stein DJ, Seedat S, Herman A, Williams DR. Non-fatal suicidal behavior among South African: Results from the South Africa stress and health study. Soc Psychiatry Psychiatr Epidemiol. 2008;43(6):454–461. 10.1007/s00127-008-0348-7 18473134 PMC2754160

[40] Muula AS, Kazembe LN, Rudatsikira E, Siziya S. Suicidal ideation and associated factors among in-school adolescents in Zambia. Tanzan Health Res Bull. 2007;9(3):202–206. 10.4314/thrb.v9i3.14331 18087900

[41] World Health Organization. Suicide in the world. Global health estimates [homepage on the Internet]. 2019 [cited 2020 Aug 5]. Available from: https://apps.who.int/iris/bitstream/handle/10665/326948/WHO-MSD-MER-19.3-eng.pdf

[42] Zhai H, Bai B, Chen L, et al. Correlation between family environment and suicidal ideation in university students in China. Int J Environ Res Public Health. 2015;12(2):1412–1424. 10.3390/ijerph120201412 25633031 PMC4344674

[43] Zhai H, Bai B, Chen L, et al. Correlation between family environment and suicidal ideation in university students in China. *Int J Environ Res Public Health*. 2015;12(2):1412–1424. 10.3390/ijerph120201412 25633031 PMC4344674

[44] Safer D. Adolescent/adult differences in suicidal behavior and outcome. Ann Clin Psychiatry. 1997;9(1):61–66. 10.3109/10401239709147774 9167837

[45] Zitzow D, Desjarlait F. A study of suicide attempts comparing adolescents to adults on a northern plains American Indian reservation. Am Indian Alsk Native Ment Health Res Monogr Ser. 1994;4:35–66. 10.5820/aian.mono04.1994.35 8205219

[46] Jordans M, Rathod S, Fekadu A, et al. Suicidal ideation and behaviour among community and health care seeking populations in five low- and middle-income countries: A cross-sectional study. Epidemiol Psychiatr Sci. 2018;27(4):393–402. 10.1017/S2045796017000038 28202089 PMC5559346

[47] Voss C, Ollmann TM, Miché M, et al. Prevalence, onset, and course of suicidal behavior among adolescents and young adults in Germany. JAMA Netw Open. 2019;2(10):e1914386. 10.1001/jamanetworkopen.2019.14386 31664450 PMC6824228

[48] Pengpid S, Peltzer K. The prevalence and correlates of suicidal ideation, plans and non-fatal suicides among 15- to 69-year-old persons in Eswatini. Behav Sci. 2020;10(11):172. 10.3390/bs10110172 PMC7696382 33182681

[49] Moazzami K, Dolmatova EV, Feurdean M. Suicidal ideation among adults with cardiovascular disease: The national health and nutrition examination survey. Gen Hosp Psychiatry. 2018;51:5–9. 10.1016/j.genhosppsych.2017.12.001 29268167

[50] Angelakis I, Gillespie EL, Panagioti M. Childhood maltreatment and adult suicidality: A comprehensive systematic review with meta-analysis. Psychol Med. 2019;49(7):1057–1078. 10.1017/S0033291718003823 30608046 PMC6498789

[51] Abdu Z, Hajure M, Desalegn D. Suicidal behavior and associated factors among students in Mettu University, South West Ethiopia, 2019: An institutional based cross-sectional study. Psychol Res Behav Manag. 2020;13:233–243. 10.2147/PRBM.S240827 32184684 PMC7061437

[52] Lengvenyte A, Conejero I, Courtet P, Olié E. Biological bases of suicidal behaviours: A narrative review. Eur J Neuroscience. 2019;53:330–351. 10.1111/ejn.14635 31793103

[53] Schlebusch L, Vawda N. HIV-infection as a self-reported risk factor for attempted suicide in South Africa. Afr J Psych. 2010;13(4):280–283. 10.4314/ajpsy.v13i4.61877 20957327

[54] Bertolote JM, Fleischmann A, De Leo D, et al. Suicidal thoughts, suicide plans and attempts in the general population on different continents. In: Wasserman D, Wasserman C, editors. Oxford textbook of suicidology and suicide prevention: A global perspective. Oxford: Oxford University Press, 2009; p. 99–104.

[55] World Health Organization. Suicide prevention (SUPRE) [homepage on the Internet]. Geneva: WHO; 2012 [cited 2020 Dec 22]. Available from: http://www.who.int/mental_health/prevention/suicide/country_reports/en/index.html

[56] Gizachew KD, Chekol YA, Basha EA, et al. Suicidal ideation and attempt among people living with HIV/AIDS in selected public hospitals: Central Ethiopia. Ann Gen Psychiatry. 2021;20:15. 10.1186/s12991-021-00335-5 33608017 PMC7896396

[57] Wang C, Pan R, Wan X, et al. Immediate psychological responses and associated factors during the initial stage of the 2019 coronavirus disease (COVID-19) epidemic among the general population in China. Int J Environ Res Public Health. 2020;17(5):1729. 10.3390/ijerph17051729 PMC7084952 32155789

[58] John A, Okolie C, Eyles E, et al. The impact of the COVID-19 pandemic on self-harm and suicidal behaviour: A living systematic review. F1000Res. 2020;9:644. 10.12688/f1000research.24274.1 PMC7871358 33604025

